# Assessing the impact of regional laboratory networks in East and West Africa on national health security capacities

**DOI:** 10.1371/journal.pgph.0001962

**Published:** 2023-05-24

**Authors:** Avery League, Donewell Bangure, Mark J. Meyer, Stephanie J. Salyer, Dorcas Wanjohi, Yenew Kebede Tebeje, Erin M. Sorrell, Claire J. Standley

**Affiliations:** 1 Milken Institute School of Public Health, The George Washington University, Washington, DC, United States of America; 2 Laboratory Systems and Networks Division, Africa Centres for Disease Control and Prevention, Addis Ababa, Ethiopia; 3 Department of Mathematics and Statistics, Georgetown University, Washington, DC, United States of America; 4 Center for Global Health Science and Security, Georgetown University, Washington, DC, United States of America; 5 Surveillance and Disease Intelligence Networks Division, Africa Centres for Disease Control and Prevention, Addis Ababa, Ethiopia; 6 Division of Global Health Protection, U.S. Centers for Disease Control and Prevention, Atlanta, Georgia; 7 Department of Microbiology & Immunology, Georgetown University, Washington, DC, United States of America; 8 Heidelberg Institute of Global Health, University of Heidelberg, Heidelberg, Germany; Burnet Institute, AUSTRALIA

## Abstract

National laboratories are a fundamental capacity for public health, contributing to disease surveillance and outbreak response. The establishment of regional laboratory networks has been posited as a means of improving health security across multiple countries. Our study objective was to assess whether membership in regional laboratory networks in Africa has an effect on national health security capacities and outbreak response. We conducted a literature review to select regional laboratory networks in the Eastern and Western African regions. We examined data from the World Health Organization Joint External Evaluation (JEE) mission reports, the 2018 WHO States Parties Annual Report (SPAR), and the 2019 Global Health Security Index (GHS). We compared the average scores of countries that are members of a regional laboratory network to those that are not. We also assessed country-level diagnostic and testing indicators during the COVID-19 pandemic. We found no significant differences in any of the selected health security metrics for member versus non-member countries of the either the East Africa Public Health Laboratory Networking Project (EAPHLNP) in the Eastern Africa region, nor for the West African Network of Clinical Laboratories (RESAOLAB) in the Western Africa region. No statistically significant differences were observed in COVID-19 testing rates in either region. Small sample sizes and the inherent heterogeneities in governance, health, and other factors between countries within and between regions limited all analyses. These results suggest potential benefit in setting baseline capacity for network inclusion and developing regional metrics for measuring network impact, but also beyond national health security capacities, other effects that may be required to justify continued support for regional laboratory networks.

## Introduction

The emergence and international spread of public health threats like the ongoing coronavirus disease 2019 (COVID-19) pandemic highlight the importance of ensuring that all countries have minimum health security capacities in place to achieve adequate preparedness and response for epidemics. Per the International Health Regulations 2005, (IHR) [1], all World Health Organization (WHO) Member States must establish core capacities to assess, detect, report, and respond to potential public health events of international concern. Core capacity areas include laboratories, surveillance, workforce, and emergency response operations.

By 2012, many countries were struggling to meet IHR requirements. These concerns, emphasized by the 2014 West Africa Ebola outbreak, led the WHO to revise the IHR Monitoring and Evaluation Framework [2]. Revisions included creating mandatory annual reporting through the State Party Self-Assessment Annual Reporting (SPAR) tool [3] and a voluntary external assessment through the Joint External Evaluation (JEE) tool, intended to be repeated every 4–5 years [4, 5].

These updates have galvanized renewed interest in innovative approaches to IHR implementation. Particularly in lower resource contexts, regional approaches to capacity building, like regional networks, are seen as a means of sharing costs across multiple countries. Sharing of resources can also limit redundancy in capacity strengthening and reduce infrastructure development and maintenance costs.

Networks can be disease- or syndrome-specific [6–8], or focused more broadly across a technical approach or area relevant to health security, such as surveillance [9], laboratories [10], or workforce development [11]. There have even been efforts to build regional networks for Emergency Operations Centers [12] to better integrate national public health emergency management capabilities into regional preparedness and response efforts. The positive impact of networks on individual diseases, and on overall regional capacity, has been well demonstrated [13–17]. However, the contribution that regional networks have on building national health security capacity, and more specifically the ability of existing IHR and other health security metrics to measure these potential contributions, has to our knowledge, rarely been demonstrated. Metrics to do so are still only in the early stages of deployment [18].

Analysis of infectious disease outbreak risk factors suggests that 22 out of the 25 most vulnerable national health systems in the world are in Africa [19]. Several of these countries concurrently receive substantial support for health security capacity strengthening from external donors, with more modest domestic public spending on health [20, 21]. African countries may particularly benefit from pooling regional resources, especially in the laboratory sector, given that public health laboratories are considered a fundamental capacity for disease surveillance and outbreak response [22]. The WHO 2008 Maputo Declaration reinvigorated support for strengthening laboratory capacity [23] in line with IHR and the WHO African Region (AFRO) Integrated Disease Surveillance and Response (IDSR) framework [24]. This resulted in efforts to promote laboratory networks across the continent [25]. Further, in 2015, the African Society for Laboratory Medicine Freetown Declaration recognized the need for multi-country and pan-African strategies to prevent and control disease outbreaks–prioritizing the strengthening of functional tiered laboratory networks [26, 27].

Here, we sought to answer the following question: does membership in regional laboratory networks in Africa have an effect on national health security capacities and outbreak response?

## Methods

### Selection of networks

An online search was conducted for eligible regional laboratory networks in Africa. To be considered eligible, a network had to meet five criteria: (1) be a formal partnership between countries that are all Member States of the African Union; (2) have three or more countries in the network; (3) be located entirely within a specific region of Africa or contiguous to it; (4) focus on general diagnostics, surveillance, and other laboratory work rather than on specific diseases; and (5) be operational with publicly available information on specific projects as of October 2019.

We identified two networks that met these criteria: the West African Network of Clinical Laboratories (RESAOLAB), and the East Africa Public Health Laboratory Networking Project (EAPHLNP). RESAOLAB is described as the first regional network in West Africa to focus on creating a system of biological laboratories offering quality services, and to improve clinical diagnosis for public health aims [28]. EAPHLNP’s objective is to “to establish a network of efficient, high quality, accessible public health laboratories for the diagnosis and surveillance of Tuberculosis and other communicable diseases” [29]. To this end, both networks are self-described as having goals that are closely aligned with global health security outcomes.

### Defining countries for inclusion

**Fig 1:**
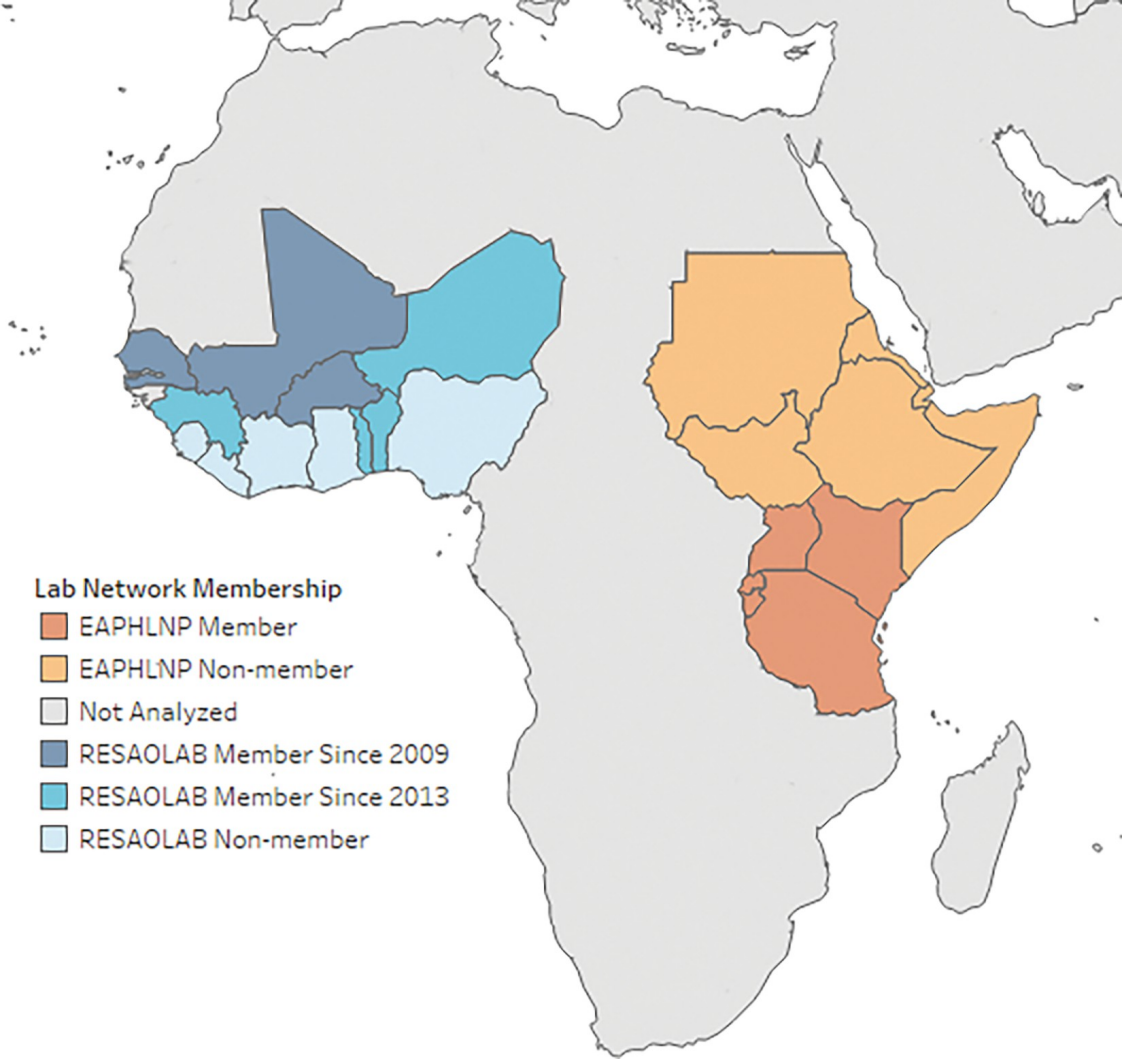
Maps of laboratory network members and non-members in the Eastern Africa (top) and Western Africa (bottom) regions. Base map layer from Natural Earth (https://www.naturalearthdata.com/http//www.naturalearthdata.com/download/50m/cultural/ne_50m_admin_0_countries.zip.) and manually edited.

Fig 1 shows the breakdown of member and non-member countries for the West African region (RESAOLAB members and non-members) and the East African region (EAPHLNP members and non-members). Non-member countries for inclusion were defined per the African Union regions; [30] however, island nations were excluded given the lack of geographical continuity with other countries in the respective regions. For the purposes of additional analyses, scores were also collated within the RESAOLAB members based on the year of membership in the network (2009 or 2013) (Fig 1).

### Health security metrics data collection and analysis

Three tools for measuring national health security capacity were used: the JEE [4], SPAR [31], and the Global Health Security Index (GHS Index). The GHS Index is a composite metric that combines elements of the JEE with indicators related to health systems, compliance with international norms, and risk environment [32].

The scores of member countries within each network, as well as non-member countries within the same region of Africa, were collated for: 1) the 48 JEE indicators of the first edition JEE tool; 2) the 13 SPAR indicators and the three *C5 Laboratory* sub-indicators for 2018; and 3) the 34 GHS Index indicators and the three *2*.*1 Laboratory Systems* sub-indicators for 2019.

The JEE indicators were additionally sorted into four categories: 1) the four *National Laboratory System* indicators (D.1.1-D.1.4); 2) *laboratory-related* indicators, which were identified in the literature review as potentially being influenced by laboratory networks or where laboratories were cited in the JEE indicator description; 3) *non-laboratory* indicators, which made no mention of laboratories in either the JEE indicator description or guiding questions; and 4) *other* indicators, which included minimal mention of laboratories or laboratory services in the JEE guiding questions or only referenced non-biological laboratories, and which were discarded from further analysis to reduce the number of multiple comparisons. The inclusion of the non-laboratory indicators was used to establish whether there were differences in overall health security capacities between groups.

Differences in the National Laboratory Systems, laboratory-related, and non-laboratory JEE indicator scores of network member countries versus non-member countries were calculated using the Goodman-Kruskal (G-K) Gamma statistic. See Supplemental Material for further description of this statistic and corresponding derivation of p-values.

Differences in the median 2018 SPAR and 2019 GHS Index scores of network member countries versus non-member countries were calculated using Kruskal-Wallis tests, with adjusted p-values for multiple comparisons. The standard deviation of each average score was also calculated, and the data used to calculate each average score were checked for outliers. Data points were considered outliers if they were more than two standard deviations away from the mean. Sample sizes were insufficient for comparisons between countries that joined RESAOLAB in 2009 versus 2013 and non-members, across all metrics.

### COVID-19 testing data collection and analysis

We also sought to assess whether membership in a regional network might affect laboratory capacities in practice, as opposed to “on paper”. To do so, we collated data provided to the Africa Centres for Disease Control and Prevention (Africa CDC) from Member States on: 1) the date at which testing capability was established in the country; 2) the number of reported severe acute respiratory syndrome coronavirus 2 (SARS-CoV-2) tests performed in the country per day; and 3) the incidence per 100,000 population of laboratory-confirmed COVID-19 cases per country. These data were reported publicly via the Africa CDC COVID-19 Dashboard [33].

To assess the effect of laboratory membership on testing capacity, we fit negative-binomial mixed effects models with number of daily tests as the outcome and laboratory membership as the primary covariate of interest. Further details on these models are available as a supplemental document (S1 Text).

## Results

### Analysis of health security metrics

The analysis of JEE scores between EAPHLNP member countries and those Eastern Africa region countries outside the network showed no significant differences across any of the analyzed indicators (Table 1). Likewise, the comparison of RESAOLAB members to non-members revealed no statistically significant differences (Table 2). Apart from network members showing higher, albeit not significantly, average score values for National Laboratory System indicators in both regions, there were no discernible patterns with respect to scores across the other analyzed indicators.

**Table 1:** JEE scores and score comparisons for the Eastern Africa region countries under consideration. The score difference was calculated as the member country average indicator score minus the non-member country average indicator score. Comparisons of the ordinal scores were made using the Goodman-Kruskal (G-K) gamma statistic; significance was tested using permutation-based, Benjamini-Hochberg (B-H) adjusted p-values. Results with significant p-values (at a 95% confidence level) are shown in bold.

JEE indicator		Indicator Summary Description	Average JEE Indicator Score (s.d.)			G-K Gamma	B-H p-value
			EAPHLNP member countries	Non-member countries	Score difference		
National Laboratory System indicators	D.1.1	Laboratory testing for priority diseases	3.60 (0.55)	3.33 (0.82)	0.27	0.29	1.00
	D.1.2	Specimen referral and transport system	2.80 (0.84)	2.33 (0.82)	0.47	0.40	0.99
	D.1.3	Point of care and laboratory diagnostics	2.60 (0.55)	2.17 (0.75)	0.43	0.56	0.86
	D.1.4	Laboratory quality system	3.00 (0.71)	1.67 (0.52)	1.33	1.00	0.58
Other indicators related to laboratory systems	P.3.1	Antimicrobial resistance (AMR) detection	1.20 (0.45)	1.33 (0.82)	-0.13	0.00	1.00
	P.5.1	Detecting and responding to foodborne disease	2.20 (0.45)	1.83 (0.41)	0.37	1.00	0.99
	P.6.1	Multisectoral biosafety and biosecurity system	2.40 (0.55)	1.50 (0.55)	0.90	1.00	0.67
	P.6.2	Biosafety and biosecurity training and practices	2.80 (0.45)	1.50 (0.55)	1.30	1.00	0.58
	D.4.1	Human resources for IHR core capacities	2.60 (0.55)	2.17 (0.75)	0.43	0.56	0.86
	D.4.3	Workforce strategy	2.50 (0.50)	2.33 (1.03)	0.17	0.16	1.00
Non-laboratory indicators	P.1.1	Legislation in place for IHR	2.00 (1.00)	2.33 (1.03)	-0.33	-0.22	1.00
	P.1.2	Domestic legislation adjusted for IHR	2.20 (1.10)	2.00 (1.10)	0.20	0.15	1.00
	P.3.2	Surveillance of AMR infections	1.20 (0.45)	1.33 (0.52)	-0.13	-0.33	1.00
	P.3.4	Antimicrobial stewardship activities	1.40 (0.89)	1.33 (0.52)	0.07	-0.14	1.00
	P.4.2	Animal Health and Veterinarian Workforce	2.00 (1.00)	2.83 (0.98)	-0.83	-0.52	0.86
	P.4.3	Mechanisms for responding to infectious zoonooses	2.40 (0.89)	1.83 (1.17)	0.57	0.44	0.86
	P.7.1	Vaccine coverage as part of a national program	3.60 (0.89)	2.67 (1.51)	0.93	0.60	0.86
	P.7.2	National vaccine access and delivery	4.20 (0.45)	3.67 (1.03)	0.53	0.53	0.86
	D.2.1*	Indicator and event-based surveillance systems	3.40 (0.89)	2.83 (0.41)	0.57	0.58	0.86
	D.2.2	Electronic real-time reporting system	2.40 (0.55)	1.67 (0.52)	0.73	1.00	0.86
	D.3.1	System for efficient reporting	2.60 (0.55)	2.33 (0.52)	0.27	0.50	0.99
	D.3.2	Reporting network and protocols in-country	2.00 (0.71)	2.00 (0.63)	0	0.00	1.00
	R.1.1	Multi-hazard national public health emergency plan	1.40 (0.55)	1.83 (0.98)	-0.43	-0.37	1.00
	R.1.2	Priority public health risks and resources are mapped and utilized	1.60 (0.55)	1.67 (1.21)	-0.07	0.26	1.00
	R.2.1	Capacity for active emergency operations	2.20 (1.10)	1.50 (0.55)	0.70	0.67	0.86
	R.2.2	Emergency Operations Centre procedures and plans	2.60 (1.14)	1.33 (0.82)	1.27	0.83	0.67
	R.2.3	Emergency operations program	2.40 (1.52)	1.83 (1.17)	0.57	0.30	1.00
	R.2.4	Case management procedures are implemented	2.00 (0.71)	2.00 (0.00)	0	0.00	1.00
	R.3.1	Public health and security authorities are linked	2.60 (1.52)	2.33 (1.51)	0.27	0.18	1.00
	R.4.2	Sending and receiving health personnel	1.40 (0.55)	2.17 (1.47)	-0.77	-0.56	0.86
	R.5.1	Risk communication systems	1.60 (0.55)	1.67 (0.82)	-0.07	0.00	1.00
	R.5.2	Internal and partner communication/coordination	3.40 (1.14)	2.67 (0.52)	0.73	0.60	0.86
	R.5.3	Public communication	3.40 (1.14)	2.50 (1.05)	0.90	0.57	0.86
	R.5.4	Communication with affected communities	3.00 (1.00)	2.50 (1.05)	0.50	0.36	0.99
	R.5.5	Dynamic listening and rumor management	2.60 (0.89)	2.33 (0.82)	0.27	0.14	1.00

*Note that in the JEE Tool second edition, this indicator was combined with D.2.4 (Syndromic surveillance systems) into the indicator D.2.1 “Surveillance systems”.

**Table 2:** JEE scores and score comparisons for the Western Africa region countries under consideration. The score difference was calculated as the member country average indicator score minus the non-member country average indicator score. Comparisons of the ordinal scores were made using the Goodman-Kruskal (G-K) gamma statistic; significance was tested using permutation-based, Benjamini-Hochberg (B-H) adjusted p-values.

JEE indicator		Indicator Summary Description	Average JEE Indicator Score (s.d.)			G-K Gamma	B-H p-value
			RESAOLAB member countries	Non-member countries	Score difference		
National Laboratory System indicators	D.1.1	Laboratory testing for priority diseases	3.43 (0.53)	2.75 (0.76)	0.68	0.74	0.56
	D.1.2	Specimen referral and transport system	2.43 (0.79)	1.83 (0.75)	0.6	0.60	0.72
	D.1.3	Point of care and laboratory diagnostics	2.86 (0.38)	2.17 (0.41)	0.69	0.94	0.49
	D.1.4	Laboratory quality system	2.14 (0.69)	1.75 (0.76)	0.39	0.38	1.00
Other indicators related to laboratory systems	P.3.1	Antimicrobial resistance (AMR) detection	1.29 (0.76)	1.17 (0.41)	0.12	0.00	1.00
	P.5.1	Detecting and responding to foodborne disease	1.57 (0.53)	1.83 (0.41)	-0.26	-0.58	1.00
	P.6.1	Multisectoral biosafety and biosecurity system	1.43 (0.53)	1.50 (0.55)	-0.07	-0.14	1.00
	P.6.2	Biosafety and biosecurity training and practices	1.57 (0.53)	1.67 (0.52)	-0.1	-0.20	1.00
	D.4.1	Human resources for IHR core capacities	2.71 (0.49)	1.83 (0.75)	0.88	0.87	0.50
	D.4.3	Workforce strategy	2.00 (0.00)	2.08 (0.49)	-0.08	-1.00	1.00
Non-laboratory indicators	P.1.1	Legislation in place for IHR	1.29 (0.49)	1.83 (0.41)	-0.54	-0.85	0.56
	P.1.2	Domestic legislation adjusted for IHR	1.29 (0.49)	1.67 (0.52)	-0.38	-0.67	0.72
	P.3.2	Surveillance of AMR infections	1.00 (0.00)	1.17 (0.41)	-0.17	-1.00	1.00
	P.3.4	Antimicrobial stewardship activities	1.00 (0.00)	1.17 (0.41)	-0.17	-1.00	1.00
	P.4.2	Animal Health and Veterinarian Workforce	2.43 (0.53)	2.17 (0.98)	0.26	0.17	1.00
	P.4.3	Mechanisms for responding to infectious zoonooses	1.86 (0.69)	1.83 (0.75)	0.03	0.04	1.00
	P.7.1	Vaccine coverage as part of a national program	2.86 (0.38)	3.33 (0.52)	-0.47	-1.00	0.72
	P.7.2	National vaccine access and delivery	3.43 (0.53)	3.50 (0.55)	-0.07	-0.14	1.00
	D.2.1*	Indicator and event-based surveillance systems	3.00 (0.00)	3.33 (0.52)	-0.33	-1.00	1.00
	D.2.2	Electronic real-time reporting system	2.14 (0.38)	2.00 (0.63)	0.14	0.29	1.00
	D.3.1	System for efficient reporting	2.71 (0.49)	2.50 (0.84)	0.21	0.20	1.00
	D.3.2	Reporting network and protocols in-country	1.71 (0.49)	2.00 (0.00)	-0.29	-1.00	1.00
	R.1.1	Multi-hazard national public health emergency plan	1.57 (0.53)	1.33 (0.52)	0.24	0.45	1.00
	R.1.2	Priority public health risks and resources are mapped and utilized	1.71 (0.49)	1.50 (0.55)	0.21	0.43	1.00
	R.2.1	Capacity for active emergency operations	1.43 (0.79)	2.33 (1.03)	-0.9	-0.70	0.56
	R.2.2	Emergency Operations Centre procedures and plans	1.14 (0.79)	2.17 (1.17)	-1.03	-0.86	0.50
	R.2.3	Emergency operations program	2.00 (0.82)	2.83 (1.17)	-0.83	-0.58	0.62
	R.2.4	Case management procedures are implemented	1.86 (0.38)	2.00 (0.00)	-0.14	-1.00	1.00
	R.3.1	Public health and security authorities are linked	1.71 (0.49)	2.33 (1.37)	-0.62	-0.29	1.00
	R.4.2	Sending and receiving health personnel	1.14 (0.38)	1.50 (1.22)	-0.36	-0.17	1.00
	R.5.1	Risk communication systems	1.00 (0.00)	2.00 (0.89)	-1	-1.00	0.50
	R.5.2	Internal and partner communication/coordination	2.00 (0.00)	3.33 (0.52)	-1.33	-1.00	0.17
	R.5.3	Public communication	2.14 (0.69)	2.83 (0.75)	-0.69	-0.71	0.60
	R.5.4	Communication with affected communities	2.43 (0.79)	2.17 (0.41)	0.26	0.38	1.00
	R.5.5	Dynamic listening and rumor management	2.14 (0.38)	2.50 (0.55)	-0.36	-0.71	0.72

*Note that in the JEE Tool second edition, this indicator was combined with D.2.4 (Syndromic surveillance systems) into the indicator D.2.1 “Surveillance systems”.

**Fig 2:**
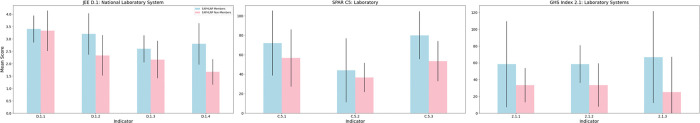
Average JEE (left), SPAR (middle) and GHS Index (right) laboratory indicator scores for EAPHLNP member (blue) and non-member (red) countries in the Eastern Africa region. Error bars represent standard deviation; significance threshold was set at p < 0.05 (none of the results were statistically significant). D.1.1 = Laboratory testing for priority diseases; D.1.2 = Specimen referral and transport systems; D.1.3 = Point of care and laboratory diagnostics; D.1.4 = Laboratory quality system; C.5.1 = Specimen referral and transport system; C.5.2 = Implementation of laboratory biosafety and biosecurity regime; C.5.3 = Access to laboratory testing for priority diseases; 2.1.1 = Laboratory capacity for detecting priority diseases; 2.1.2 = Specimen referral and transport system; 2.1.3 = Laboratory quality systems.

**Fig 3:**
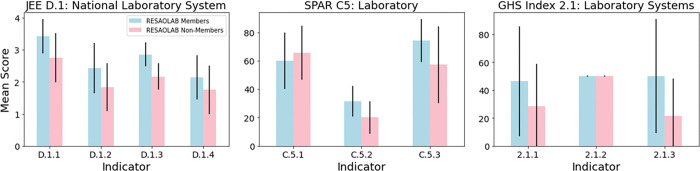
Average JEE (left), SPAR (middle) and GHS Index (right) laboratory indicator scores for RESAOLAB member (blue) and non-member (red) countries in the Western Africa region. Error bars represent standard deviation; significance threshold was set at p < 0.05 (none of the results were statistically significant). D.1.1 = Laboratory testing for priority diseases; D.1.2 = Specimen referral and transport systems; D.1.3 = Point of care and laboratory diagnostics; D.1.4 = Laboratory quality system; C.5.1 = Specimen referral and transport system; C.5.2 = Implementation of laboratory biosafety and biosecurity regime; C.5.3 = Access to laboratory testing for priority diseases; 2.1.1 = Laboratory capacity for detecting priority diseases; 2.1.2 = Specimen referral and transport system; 2.1.3 = Laboratory quality systems.

All three SPAR and all three GHS Index laboratory indicators for both EAPHLNP and RESAOLAB showed no statistically significant difference against their respective non-members (Figs 2 and 3). There was no consistent pattern, and no significant differences, between EAPHLNP or RESAOLAB members and non-members for the non-laboratory JEE, SPAR and GHS Index indicators (Tables 1 and 2, S1 and S2 Tables). Ethiopia, not an EAPHLNP member, was a high outlier across three 2019 GHS Index indicators (Biosafety [1.4], IHR Reporting Compliance and Disaster Risk Reduction [5.1], and Cross-Border Agreements on Public and Animal Health Emergency Response [5.2]), scoring higher than other non-member countries in East Africa. Nigeria, a non-RESAOLAB member, was a high outlier for two 2018 SPAR indicators (Legislation and Financing [C1] and Food Safety [C4]) and one GHS Index indicator (Biosecurity [1.3]), scoring higher than other non-network members in the Western Africa region.

### COVID-19 testing data analysis

**Fig 4:**
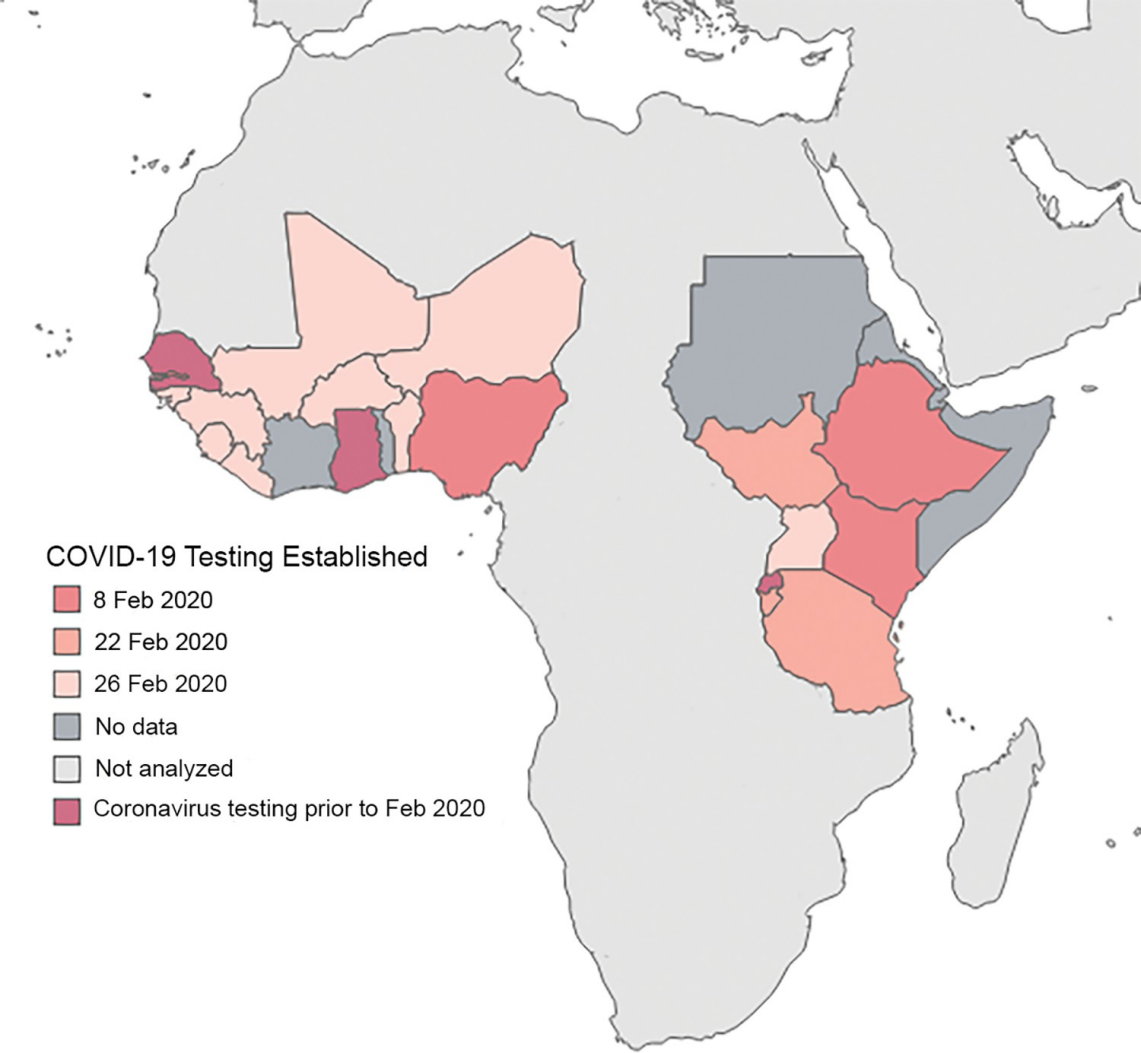
Date COVID-19 testing established in countries included in the analyses. Base map layer from Natural Earth (https://www.naturalearthdata.com/http//www.naturalearthdata.com/download/50m/cultural/ne_50m_admin_0_countries.zip.) and manually edited.

Fig 4 shows the date that testing capacity was established in each of the countries included in our analyses.

Our model showed no impact of laboratory network membership on testing rate while controlling for daily incidence of reported COVID-19 cases and time since the first reported test in both networks (S3 Table). The EAPHLNP member countries reported more tests conducted than non-member countries, but the difference did not reach statistical significance. There was an insufficient number of countries with testing capability established prior to the COVID-19 pandemic to include this as a variable in the model.

## Discussion

The lack of statistically significant differences in any of the analyzed health security framework indicators suggests a number of possible interpretations. Despite member countries having numerically higher average scores across most laboratory-related indicators across all three metrics, the small sample sizes limited the power of our statistical analyses, in both regions. This suggests that other approaches that employ qualitative methods might be better suited to understanding potential differences with small samples, in order to further understand the impact of networks in these regions. The small sample sizes also may have overrepresented outliers. Non-network members, Nigeria and Ethiopia, in the Western and Eastern Africa regions respectively, were both high outliers across different SPAR or GHS Index indicators. Neither are network members, but both were priority countries for United States Global Health Security Agenda (GHSA) assistance, of which laboratory capacity strengthening was a core activity. Other countries in the two regions did also benefit from GHSA investment, and may have received other types of bilateral or multilateral assistance. Future analyses of the effect of laboratory networks on national capacities should therefore attempt to consider large-scale assistance and cooperation programs as potential competing or confounding variables.

Our analysis of COVID-19 testing data showed no statistically significant relationship between network membership and testing rates. The analysis was limited by focusing only on COVID-19 testing data. A more robust test of national laboratory network resilience would have also measured diagnostic activity for non-COVID suspected specimens (i.e. HIV viral load testing), to ensure that the scale-up of COVID-19 testing did not come at the expense of other laboratory activities. Similarly, regional entities, including the Africa CDC, have provided substantial targeted support for various aspects of COVID-19 diagnostic testing since the literature review for this paper took place, as have other bilateral and multilateral donors. Africa CDC support has included the provision of training, supplies, reagents, and web-based resources, any of which could have influenced testing rates independent of network membership [34, 35]. Future studies could also consider analyzing notifications and alerts related to public health events prior to COVID-19, to identify a potential link between laboratory reactiveness and network membership; however, databases of outbreak alerts have known limitations, which would need to be addressed before a comprehensive analysis of this sort could be undertaken [36].

Our results provide no evidence of a beneficial effect of regional laboratory networks on national level laboratory-related health security capacities, at least based on the metrics analyzed here. It is worth highlighting that we did not attempt to analyze the existence of baseline capacity inclusion criteria or the success of the networks, based on their own stated goals and objectives; our analysis was limited to the potential influence of network membership on national health security indicators. We also did not seek to examine potential regional level benefits; to our knowledge, such regional level indicators do not yet exist within the context of global health security frameworks. The indicators used by RESAOLAB and EAPHLNP themselves were largely applied at the national, rather than regional, level, but could constitute a starting point for development of relevant regional indicators for laboratory systems, for example with respect to proportion of disease events that receive confirmatory testing, irrespective of whether the test is performed within the same country or another country in the network [37, 38].

There are numerous examples of efforts to establish regional laboratory networks from regions outside of Africa [39–41]. Like RESAOLAB and EAPHLNP, these networks have received widespread interest and promote open communication and data sharing [42–44]. Despite this, to our knowledge, there have been no analyses to date to demonstrate or quantify the potential benefits of membership in these types of networks in terms of national compliance with health security frameworks, or regional capabilities. To this end, our research adds to this area of scholarship from a methodological perspective and suggests there may be opportunities, especially in world regions where multi-country political or economic coalitions are already in place and functional, to complement existing nationally-focused health security metrics with indicators that capture capacities at a regional level as well.

The ongoing COVID-19 pandemic has highlighted the importance of improving core capacities, like laboratory diagnostics, in health security systems [45]. The pandemic has emphasized the need for scalable, rapidly-activated diagnostic testing platforms, strengthened global reagent supply chains, and sufficient trained laboratory personnel in countries worldwide. While there is an ongoing debate about the value of metrics such as JEE, SPAR, and GHS Index scores in predicting response outcomes at national levels [46–49], there may yet be further potential in leveraging regional initiatives to support national health security capacities [50]. In Africa for example, additional laboratory networks are being established to strengthen health security like the Institute of Pathogen Genomics (IPG) laboratory network. IPG operates through Africa CDC’s Regional Coordinating Centers and its Regional Integrated Surveillance and Laboratory Network (RISLNET) [51] and has already shown great success in establishing regional and continental genomics capacity for COVID-19 that can be leveraged for future pathogen discovery [52–54]. Additionally, Africa CDC and the African Union has called for African countries to pool resources to procure and distribute COVID-19 diagnostic tests, allowing for greater national-level access to testing [55]. This kind of sharing of resources can be further facilitated through regional health security systems, including regional laboratory networks such as RESAOLAB and EAPHLNP. Such benefits could be captured through new regional health security metrics, as described above, as a means of more accurately characterizing the benefits of regional networks.

Our study had numerous limitations. It cannot be ascertained whether the scores on laboratory-related indicators reflected membership in the regional networks, a self-selection bias, legacies of pre-membership laboratory capacity status, one or more with unmeasured factors, or a confounding factor. Individual country-level variation in size, political attention or priority given to health security, access to resources, and other bilateral and multilateral relationships and partnerships outside of the networks may influence laboratory capacity, as well as the ability to benefit from networks. Numerous commentators have questioned the value of health security metrics as measures of laboratory and other essential public health capacities. While we attempted to address this limitation by including testing rate data, numerous other factors could have contributed to testing capacity. These include domestic attention to testing as a core response strategy and bilateral or multilateral support from non-network sources.

## Conclusion

Our study showed no statistically significant benefits of membership in regional laboratory networks on national-level laboratory capacities, in either studied region, nor on any of the other non-laboratory health security indicators analyzed. The lack of observed benefit across these indicators may in part reflect imprecision in the original indicators, the methodological approach, or shortcomings of indicators as a proxy for capacity. Taken at face value, it could be suggested that national health security capacities are, as a stand-alone outcome, insufficiently addressed by these regional networks. However, our analysis did not directly consider network inclusion criteria or other stated goals and outcomes of the selected networks; for example, improved quality of services, which could itself justify continued support for regional laboratory networks. Moreover, there may be opportunities to focus on development of regional capacity indicators as a promising and important area for future exploration.

## Supporting information

S1 Table Table comparing member and non-member countries in EAPHLNP and RESAOLAB in the Eastern and Western Africa regions, respectively, by SPAR average scores, standard deviations, and adjusted p-values for all non-laboratory indicators. (DOCX)

S2 Table Table comparing member and non-member countries in EAPHLNP and RESAOLAB in the Eastern and Western Africa regions, respectively, by GHI Index average scores, standard deviations, and adjusted p-values for all non-laboratory indicators. (DOCX)

S3 Table Results of the comparison of SARS-CoV-2 testing rates between network member and non-member countries in West and East African regions. (DOCX)

S1 Text Additional description of statistical methods used for comparison of Joint External Evaluation indicator scores and COVID-19 testing data. (DOCX)
