# Supplementary material for: Assessing the impact of regional laboratory networks in East and West Africa on national health security capacities
Source: PLOS Glob Public Health. 2023 May 24;3(5):e0001962. doi: 10.1371/journal.pgph.0001962 (PMC10208488; doi:10.1371/journal.pgph.0001962)
Supplement: S1 Table — (DOCX) [file pgph.0001962.s001.docx]

**S1 Table. SPAR Indicators**

|  | **West Africa: Average Scores**  **(± Standard Deviation)** | | | | | **East Africa: Average Scores**  **(± Standard Deviation)** | | |  |
| --- | --- | --- | --- | --- | --- | --- | --- | --- | --- |
| **Indicator** | **RESAOLAB Members** | **RESAOLAB**  **Non-members** | | **Adjusted**  **p-value** | | **EAPHLNP Members** | **EAPHLNP Non-Members** | **Adjusted p-value** |  |
| **Laboratory indicator** | | | | | | | | |  |
| **C5** | 55.29 (±13.11) | | 47.71 (±16.92) | | 0.75 | 65.20 (±23.35) | 49.00 (±18.15) | 0.85 | |
| **Non-laboratory indicators** | | | | | | | | |  |
| **C1** | 38.14 (±13.79) | 34.29 (± 11.73) | | 0.75 | | 44.00 (± 33.30) | 35.67 (± 21.84) | 0.93 |  |
| **C2** | 51.43 (± 15.74) | 51.43 (± 26.73) | | 0.75 | | 50.00 (± 27.39) | 56.67 (± 17.51) | 0.93 |  |
| **C3** | 54.29 (± 27.60) | 51.43 (± 15.74) | | 0.75 | | 52.00 (± 22.80) | 53.33 (± 20.66) | 0.93 |  |
| **C4** | 45.71 (± 15.12) | 34.29 (± 22.25) | | 0.64 | | 44.00 (± 26.08) | 40.00 (± 30.98) | 0.93 |  |
| **C6** | 60.00 (± 14.14) | 65.71 (± 19.02) | | 0.75 | | 58.00 (± 16.43) | 70.00 (± 18.97) | 0.85 |  |
| **C7** | 54.29 (± 22.25) | 48.57 (± 22.68) | | 0.75 | | 40.00 (± 24.49) | 56.67 (± 23.38) | 0.85 |  |
| **C8** | 44.71 (± 17.75) | 40.86 (± 10.40) | | 0.75 | | 40.00 (± 19.72) | 44.33 (± 31.58) | 0.93 |  |
| **C9** | 30.29 (± 13.76) | 35.14 (± 6.44) | | 0.75 | | 44.00 (± 16.09) | 46.67 (± 14.61) | 0.93 |  |
| **C10** | 31.43 (± 15.74) | 48.57 (± 25.45) | | 0.64 | | 40.00 (± 40.00) | 46.67 (± 27.33) | 0.93 |  |
| **C11** | 24.29 (± 21.49) | 38.57 (± 12.15) | | 0.64 | | 30.00 (± 14.14) | 31.67 (± 7.53) | 0.93 |  |
| **C12** | 22.86 (± 7.56) | 34.29 (± 29.92) | | 0.75 | | 40.00 (± 28.28) | 20.00 (± 12.65) | 0.85 |  |
| **C13** | 22.86 (± 7.56) | 31.43 (± 15.74) | | 0.75 | | 32.00 (± 17.89) | 23.33 (± 15.06) | 0.85 |  |
