# Supplementary material for: Assessing the impact of regional laboratory networks in East and West Africa on national health security capacities
Source: PLOS Glob Public Health. 2023 May 24;3(5):e0001962. doi: 10.1371/journal.pgph.0001962 (PMC10208488; doi:10.1371/journal.pgph.0001962)
Supplement: S2 Table — (DOCX) [file pgph.0001962.s002.docx]

**S2 Table. 2019 GHS Index Indicators**

|  | **West Africa: Average Scores**  **(± Standard Deviation)** | | | **East Africa: Average Scores**  **(± Standard Deviation)** | | |
| --- | --- | --- | --- | --- | --- | --- |
| **Level** | **RESAOLAB Members** | **RESAOLAB**  **Non-members** | **Adjusted p-value** | **EAPHLNP Members** | **EAPHLNP Non-Members** | **Adjusted**  **p-value** |
| **Laboratory Indicator** | | | | | | |
| **2.1** | 42.86 (± 18.27) | 33.34 (± 18.61) | 0.98 | 58.32 (± 20.41) | 30.58 (±20.19) | 0.30 |
| **Non-Laboratory Indicators** | | | | | | |
| **1.1** | 15.46 (± 22.26) | 26.19 (± 29.44) | 1.00 | 51.66 (± 13.70) | 25.00 (± 34.55) | 0.47 |
| **1.2** | 20.97 (± 16.87) | 18.56 (± 16.95) | 0.98 | 35.34 (± 18.20) | 11.60 (± 11.58) | 0.30 |
| **1.3** | 1.14 (± 1.95) | 5.14 (± 8.55) | 0.98 | 4.80 (± 8.67) | 7.33 (± 11.43) | 1.00 |
| **1.4** | 0.00 (± 0.00) | 0.00 (± 0.00) | 1.00 | 20.00 (± 27.39) | 4.17 (± 10.21) | 0.69 |
| **1.5** | 0.00 (± 0.00) | 0.00 (± 0.00) | 1.00 | 0.00 (± 0.00) | 0.00 (± 0.00) | 1.00 |
| **1.6** | 86.11 (± 10.72) | 75.07 (± 22.54) | 1.00 | 90.18 (± 11.36) | 84.65 (± 11.27) | 0.69 |
| **2.2** | 27.86 (± 16.51) | 44.30 (± 23.82) | 0.81 | 32.32 (± 28.81) | 12.50 (± 12.99) | 0.47 |
| **2.3** | 46.43 (± 9.45) | 67.86 (± 23.78) | 0.81 | 70.00 (± 44.72) | 29.17 (± 24.58) | 0.47 |
| **2.4** | 14.29 (± 37.80) | 0.00 (± 0.00) | 1.00 | 0.00 (± 0.00) | 0.00 (± 0.00) | 1.00 |
| **3.1** | 5.36 (± 6.68) | 11.61 (± 11.66) | 0.98 | 3.76 (± 5.60) | 5.22 (± 10.01) | 1.00 |
| **3.2** | 28.57 (± 39.34) | 21.43 (± 39.34) | 1.00 | 60.00 (± 22.36) | 8.33 (± 20.41) | 0.30 |
| **3.3** | 28.54 (± 12.59) | 28.54 (±12.59) | 1.00 | 13.32 (± 18.24) | 11.10 (± 17.20) | 1.00 |
| **3.4** | 0.00 (± 0.00) | 0.00 (± 0.00) | 1.00 | 20.00 (± 44.72) | 0.00 (± 0.00) | 0.79 |
| **3.5** | 17.86 (± 12.20) | 35.71 (± 37.80) | 1.00 | 45.00 (± 27.39) | 33.33 (± 43.78) | 0.69 |
| **3.6** | 56.30 (± 12.20) | 61.93 (± 8.05) | 0.98 | 53.42 (± 6.40) | 51.10 (± 8.76) | 1.00 |
| **3.7** | 100.00 (± 0.00) | 100.00 (± 0.00) | 1.00 | 90.00 (± 22.36) | 100.00 (± 0.00) | 0.79 |
| **4.1** | 4.36 (± 6.37) | 6.77 (± 8.20) | 0.81 | 9.00 (± 10.41) | 13.05 (± 17.41) | 0.53 |
| **4.2** | 14.27 (± 17.80) | 14.27 (± 17.80) | 1.00 | 6.66 (± 14.89) | 22.20 (± 17.20) | 0.53 |
| **4.3** | 31.30 (± 7.30) | 37.10 (± 17.86) | 1.00 | 35.22 (± 9.65) | 19.58 (± 10.76) | 0.30 |
| **4.4** | 7.14 (± 18.90) | 7.14 (± 18.90) | 1.00 | 0.00 (± 0.00) | 0.00 (± 0.00) | 1.00 |
| **4.5** | 0.00 (± 0.00) | 7.14 (± 18.90) | 1.00 | 0.00 (± 0.00) | 0.00 (± 0.00) | 1.00 |
| **4.6** | 10.71 (± 19.67) | 50.00 (± 28.87) | 0.81 | 45.00 (± 20.92) | 25.00 (± 38.73) | 0.47 |
| **5.1** | 57.14 (±18.90) | 57.14 (± 18.90) | 1.00 | 70.00 (± 27.39) | 58.33 (± 20.41) | 0.77 |
| **5.2** | 21.43(± 26.73) | 35.71 (± 24.40) | 0.98 | 30.00 (± 27.39) | 8.33 (± 20.41) | 0.59 |
| **5.3** | 37.49 (± 28,29) | 38.83 (± 26.07) | 1.00 | 58.12 (± 29.86) | (25.53± 27.99) | 0.37 |
| **5.4** | 50.00 (± 35.36 | 42.86 (± 18.90) | 1.00 | 40.00 (± 13.69) | 33.33 (± 12.91) | 0.72 |
| **5.5** | 50.00 (± 0.00) | 57.14 (± 13.11) | 0.98 | 50.00 (± 0.00) | 41.67 (± 13.94) | 0.69 |
| **5.6** | 66.70 (± 0.00) | 66.70 (± 0.00) | 1.00 | 66.70 (± 0.00) | 66.70 (± 0.00) | 1.00 |
| **6.1** | 47.99 (± 20.08) | 52.04 (± 24]) | 1.00 | 41.44 (± 19.32) | 22.62 (± 12.94) | 0.47 |
| **6.2** | 44.44 (± 14.86) | 43.41 (± 9.33) | 1.00 | 46.10 (± 8.94) | 38.42 (± 7.94) | 0.62 |
| **6.3** | 26.17 (± 19.49) | 23.81 (± 14.79) | 1.00 | 23.32 (± 10.86) | 26.40 (± 25.52) | 1.00 |
| **6.4** | 66.26 (± 9.24) | 57.11 (± 11.25) | 0.81 | 68.88 (± 4.90) | 54.25 (± 11.87) | 0.30 |
| **6.5** | 21.66 (± 13.31) | 17.81 (± 5.01) | 1.00 | 17.90 (± 6.21) | 13.73 (± 8.88) | 0.69 |
