# Supplementary material for: Assessing the impact of regional laboratory networks in East and West Africa on national health security capacities
Source: PLOS Glob Public Health. 2023 May 24;3(5):e0001962. doi: 10.1371/journal.pgph.0001962 (PMC10208488; doi:10.1371/journal.pgph.0001962)
Supplement: S3 Table — (DOCX) [file pgph.0001962.s003.docx]

**S3 Table: Results of the comparison of SARS-CoV-2 testing rates between network member and non-member countries in West and East African regions.** Values show the estimated regression coefficient effects from a negative-binomial mixed effects model, 95% confidence intervals, and p-values for the Eastern and Western Africa region countries under consideration. Coefficients are interpretable on the log rate ratio scale.

|  | West Africa | | East Africa | |
| --- | --- | --- | --- | --- |
| Coefficient | **Effect (95% CI)** | **p-value** | **Effect (95% CI)** | **p-value** |
| Intercept | 7.96 (6.82, 9.10) | < 0.001 | 7.47 (6.58, 8.36) | < 0.001 |
| Lab Network Member | -0.12 (-1.13, 0.88) | 0.812 | 1.36 (-0.05, 2.76) | 0.059 |
| Incidence | 0.01 (0.01, 0.01) | < 0.001 | 0.00 (0.00, 0.00) | < 0.001 |
| Time | 0.01 (0.01, 0.02) | < 0.001 | 0.02 (0.01, 0.02) | < 0.001 |
