# Supplementary material for: Assessing the impact of regional laboratory networks in East and West Africa on national health security capacities
Source: PLOS Glob Public Health. 2023 May 24;3(5):e0001962. doi: 10.1371/journal.pgph.0001962 (PMC10208488; doi:10.1371/journal.pgph.0001962)
Supplement: S1 Text — (DOCX) [file pgph.0001962.s004.docx]

**S1 Text – Additional information on statistical methods used.**

***Analysis of JEE scores***

As stated in the main manuscript, differences in the National Laboratory Systems, laboratory-related, and non-laboratory JEE indicator scores of network member states versus non-member states were calculated using the Goodman-Kruskal (G-K) Gamma statistic. The G-K Gamma measures the strength of an association between two categorical variables where one or both are ordinal. For this analysis, the JEE indicator levels are the ordinal variates as laboratory membership is binary. Values near zero suggest independence, and values close to 1 or -1 imply a lack of independence. Due to the small sample size of countries, the large-sample p-value described in Goodman and Kruskal [33] could not be invoked. Instead, we implemented a permutation test with p-values based on 100,000 resamples. Within each region, we adjusted for multiple comparisons using the standard Benjamini-Hochberg false discovery rate (FDR) procedure [34]. Due to the discrete nature of the permutation test, p-values of exactly 0 and exactly 1 are possible and realized, with p<0.05, and including 0, corresponding to a significant result.

***Analysis of COVID-19 testing incidence***

To assess the effect of laboratory membership on testing capacity, we fit negative-binomial mixed effects models with number of daily tests as the outcome, with laboratory membership was the primary covariate of interest, but the models were also adjusted for the daily incidence rate of COVID-19 (per 100,000 population) and the time (in days) since the first tests were reported to the African Union. Negative-binomial mixed effects models are longitudinal models for counting data that is over-dispersed. The models also included random country-specific intercepts and random country-specific slopes to control for within-country variation over time.
